# 

**Published:** 1895-12-07

**Authors:** 


### CONTENTS.

**The Holloway Sanatorium** - 159

**Doctors versus Drink** - 160

**On Modern Progress in Ophthalmic Medicine and Surgery.** By R. B. Carter, F.R.C.S.—Squint (*continued*) - 161

**The Vindication of Water** - 162

**Medical Progress and Hospital Clinics—**

Some Effects of Tight Lacing on Certain of the Abdominal Contents. By J. C. Webster, M.D. - 165

Progress in Medicine.—Foods and Nutrition - 166

Progress in Surgery.—General Surgery (*continued*) - 168

**Notes and News** - 16[illegible]

**Annotations.**—The Pasteurisation of Milk—The Medical Council and the Medical Profession—The Health Work of a City Company - 163

**The General Medical Council** - 171

**The Institutional Workshop—**

Hospital Construction.—The Strasburg Hospital for Women 171

Hospital Meetings - 172

The Epileptic Colony at Chalfont St. Peter - 172

The Rotunda Hospital, Dublin - 1[illegible]2

Construction Notes - 173

**Scraps and Gleanings** - 173

### EXTRA SUPPLEMENT.—THE NURSING MIRROR.

**News from the Nursing World.**—Our Needlework Competition—Nurses for Colonists—Well-merited Gratitude—Pershore Cottage Hospital—Crewe Memorial Hospital—Duty or Drudgery?—Wallsend Nursing Association—A New Association—St. Lawrence's Catholic Home—A Nurses' Home at Dundee—Queen's Nurses at Inverness—More Registration for Nurses—Paupers as Probationers—Short Items - lxzv

**Elementary Physiology for Nurses.** By C. F. Marshall, late Surgical Registrar, Hospital for Sick Children, Great Ormond Street. XVIII.—The Eye (*continued*) - lxxvii

**Where to Go** - lxxvii

**The Comforts of Nurses** - lxxviii

**The Princess Maud Marriage Present Fund** - lxxix

**Nursing in Florence** - lxxix

**The Royal British Nurses Association** - lxxix

**Everybody's Opinion** - lxxx

**The Medical Study for Women in Russia** - lxxxi

**Notes and Queries** - lxxxi

**Dundee Roynl Lunatic Asylum** - lxxxii

**For Reading to the Sick** - lxxxii

